# ﻿Review of the genus *Apiocephalus* Gahan, 1898 (Coleoptera, Cerambycidae) with description of a new species

**DOI:** 10.3897/zookeys.1226.139148

**Published:** 2025-02-06

**Authors:** Guanglin Xie

**Affiliations:** 1 Institute of Entomology, College of Agriculture, Yangtze University, Jingzhou, Hubei, 434025, China Yangtze University Jingzhou China

**Keywords:** China, identification key, Lepturinae, longicorn beetle, new record, new species, Rhagiini, taxonomy

## Abstract

Taxonomic notes on the genus *Apiocephalus* Gahan, 1898 are provided. The genus is newly recorded in China with the description of a new species, *Apiocephalusyangmingae***sp. nov.**, from the Qinling Mountains. Brief redescriptions of *Apiocephaluspunctipennis* Gahan, 1898 and *Apiocephaluslicheneus* Gahan, 1906 are presented, along with their holotype photographs. A key to the known species is given.

## ﻿Introduction

The genus *Apiocephalus* Gahan, 1898 was established for a new African species, *Apiocephaluspunctipennis* Gahan, 1898. A second species, *Apiocephaluslicheneus* Gahan, 1906, was later described from Dehradun, India. Currently, it is preliminarily placed in the tribe Rhagiini Kirby, 1837 within the subfamily Lepturinae (Švácha and Lawrence 2014) and comprises only the two aforementioned species. The genus is closely related to *Capnolymma* Pascoe, 1858 and *Acapnolymma* Gressitt & Rondon, 1970 (Švácha and Lawrence 2014). The main differences from *Capnolymma* are that the anterior part of the head is significantly shorter, the antennal insertions are more widely separated, the pronotum is furnished with four discal protuberances, and metatarsomere 1 is not longer than the next two combined. Compared to *Acapnolymma*, the genus differs primarily by the presence of pronounced lateral spines and a pair of prominent posterior discal ridges, as well as the absence of a median carina on the pronotum.

Several years ago, an intriguing individual was captured on an unidentified flower in the Qinling Mountains of Shaanxi, China. Initially, the author identified it as a member of the genus *Capnolymma* rather than *Acapnolymma* (both of which have been recorded in Yunnan, China), due to its distinct lateral spines on the pronotum. It was not until later, after examining the type specimens and other material of *Capnolymma* and *Apiocephalus* housed in the Natural History Museum, London, that the author realized it should be placed in *Apiocephalus* and represented an undescribed species. This discovery provided the author with the opportunity to conduct a brief review of this genus.

## ﻿Material and methods

Specimens from the following collections were examined and photographed in this study. The place where the specimens were deposited is indicated in the text.

**NHMUK**Natural History Museum, London, UK;

**YZU** Insect Collection, College of Agriculture, Yangtze University, Jingzhou, China.

Photographs were taken using a Canon 7D Mark II DSLR camera with a Canon EFS 100 mm lens and edited using Adobe Photoshop (2020 release). Extended depth of field at magnification was achieved by stacking multiple images from different focal planes using Helicon Focus software.

Label text for all studied specimens is reproduced verbatim, without corrections or additions, and is presented in single quotation marks. Individual labels are separated by a semicolon, and data on different rows are separated by a single slash. Additional and explanatory comments by the author are provided in square brackets. Abbreviations are used in the text for label text: “h” for handwritten, “p” for printed.

## ﻿Results

In this work, the genus *Apiocephalus* was briefly reviewed. A new species, *Apiocephalusyangmingae* sp. nov., from Shaanxi Province, China, is described and illustrated. Holotype photographs of *Apiocephaluspunctipennis* and *A.licheneus* are provided, along with a key for distinguishing these three species.

### ﻿Genus Apiocephalus

Taxon classificationAnimaliaColeopteraCerambycidae

Gahan, 1898

E9158DCA-96A4-56B7-8589-24DCE6A83D8A

Apiocephalus Gahan, 1898: 42; Gahan 1906: 74 [redescription].

#### Type species.

*Apiocephaluspunctipennis* Gahan, 1898.

#### Redescription.

Head prolonged, markedly narrowed behind eyes; eyes prominent, coarsely faceted. Antennae longer than body; scape long and curved, thickened apically; antennomere 3 approximately equal in length to antennomere 4 but distinctly shorter than antennomere 5; antennomeres 5–10 gradually decreasing in length. Pronotum strongly narrowed anteriorly, constricted before apex, with two developed lateral spines; disc provided with four protuberances, of which the anterior two are before middle, low, and the posterior two are after middle, strongly ridged. Elytra much broader than pronotum, with sides subparallel in basal two-thirds, thence converging more strongly towards apices which are truncate. Male metatarsomere 1 approximately as long as metatarsomere 2 and 3 combined.

#### Distribution.

Kenya, India, China (new country record).

#### Remarks.

This genus was first found in “British East Africa” (now part of Kenya). The second species was discovered in northern India. The third species, to be described below, comes from central China. This scattered distribution suggests that the genus exhibits a continental distribution pattern. It also indicates that with further research, additional new taxa may be discovered across the continents of Africa, Europe and Asia.

### ﻿ Apiocephalus punctipennis

Taxon classificationAnimaliaColeopteraCerambycidae

Gahan, 1898

7CDE5F9F-B185-5A0F-8022-AD4636088604

**Figure 1.**
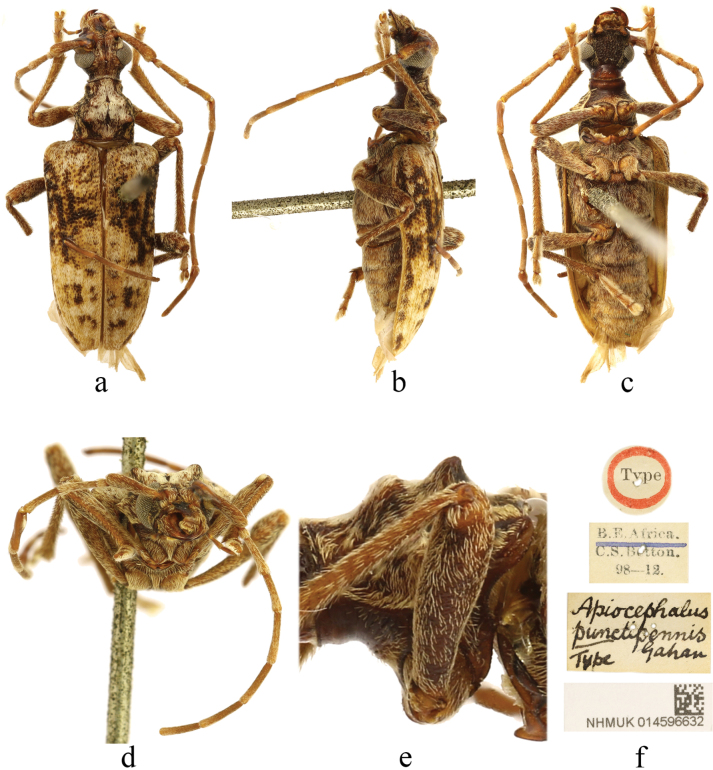
Holotype of *Apiocephaluspunctipennis* Gahan, 1898 **a** dorsal view **b** lateral view **c** ventral view **d** frontal view **e** lateral view of pronotum **f** labels.

Chinese common name: 头花天牛 Fig. 1

Apiocephalus punctipennis Gahan, 1898: 43. Type locality: Kenya (British East Africa); Aurivillius 1912: 177 [distribution]; Corinta Ferreira and Veiga Ferreira 1959 [catalogue]; Duffy 1968: 67 [larva].

#### Redescription.

**Female.** Body testaceous to blackish-brown, clothed with off-white, yellowish-brown to greyish-yellow pubescence, with glabrous areas forming black spots. Pronotum clothed with greyish-white pubescence on middle of disc. Elytra clothed with pale yellowish-brown mixed with greyish-white pubescence and decorated with glabrous irregular black patches. Head rugosely punctate, with a median sulcus on vertex and occiput. Pronotum slightly wider than long between lateral spines, at about apical third distinctly constricted, with a coniform spine on middle of each side; anterior two protuberances on disc with rather steep anterior slope. Elytra about 1.8 times as long as humeral width, with sides slightly constricted at the basal third; disc coarsely punctate, with the punctures gradually becoming shallower towards apex. Legs moderately long, with femur slightly clavate.

#### Type material examined.

***Holotype*** • female (NHMUK): ‘Type [p, label circular, red framed]; B. E. Africa. / C. S. Betton. / 98–12. [p]; Apiocephalus / punctipennis / Type / Gahan [h]; NHMUK014596632 [p]’.

#### Distribution.

Kenya.

#### Remarks.

Although Gahan (1898) did not specify the gender of the type specimen in the original description, its antennal length and abdominal shape indicate that it is female. Additionally, based on the data uploaded by Bezark (2013) on the website “Cerambycoidea”, this species occurs in Manyara, Tanzania.

### ﻿ Apiocephalus licheneus

Taxon classificationAnimaliaColeopteraCerambycidae

Gahan, 1906

0A02FBDE-6A16-5157-8A46-831087BD7ADE

**Figure 2.**
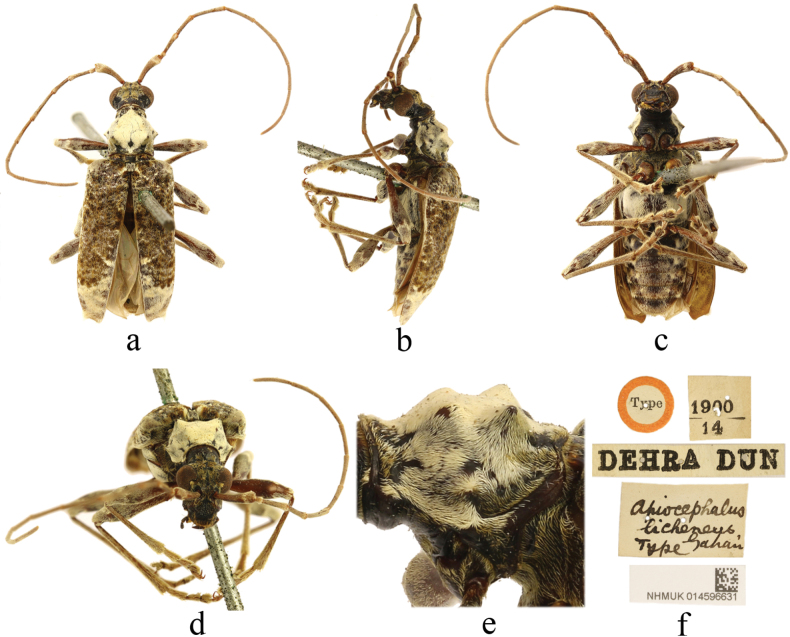
Holotype of *Apiocephaluslicheneus* Gahan, 1906 **a** dorsal view **b** lateral view **c** ventral view **d** frontal view **e** lateral view of pronotum **f** labels.

Chinese common name: 梨头花天牛 Fig. 2

Apiocephalus licheneus Gahan, 1906: 74. Type locality: India (Dehradun); Aurivillius 1912: 177 [distribution]; Duffy 1968: 67 [hosts]; Mukhopadhyay and Biswas 2011: 82 [distribution]; Mamlayya et al. 2014: 5494 [distribution]; Bhawane et al. 2015: 680 [distribution]; Kariyanna et al. 2017: 257 [distribution].

#### Redescription.

**Male.** Body reddish-brown to blackish-brown, clothed with greyish-white, greyish-yellow and blackish-brown pubescence. Greyish-white pubescence denser on apex of scape, pronotum, elytral humeri, apical fourth of elytra, apex of femora, sides of mesosternum and metepisternum. Pronotum provided with three glabrous black spots at base, of which the middle one near middle is small. Elytra mostly clothed with blackish-brown mixed greyish-yellow pubescence on basal three-fourths, irregularly interspersed with greyish-white pubescent spots. Abdominal ventrites dotted with greyish-white pubescent spots on both sides. Head slightly rugose-punctate, with a pair of small tubercles on vertex between the eyes. Pronotum slightly wider than long between lateral spines, distinctly constricted at about apical fifth, with a coniform spine on middle of each side; anterior two protuberances on disc with anterior slope relatively gentle. Elytra about 1.9 times as long as humeral width, with sides subparallel in basal two-thirds, thence converging more strongly towards apices; surface strongly and closely punctate on basal three-fourths, inconspicuously on apical fourth. Legs long, femora slightly clavate, somewhat constricted apically.

#### Type material examined.

***Holotype*** • male (NHMUK): ‘Type [p, label circular, red framed]; DEHRA DUN [p]; 1900 / 14 [p]; apiocephalus / licheneus, / Type Gahan [h]; NHMUK014596631 [p]’.

#### Distribution.

India.

#### Remarks.

This species differs from *A.punctipennis* primarily by the denser greyish-white pubescence on the pronotum, the less steep anterior slope of the two anterior protuberances on the disc of the pronotum, and the presence of pubescent black spots on the elytra instead of glabrous black spots.

### ﻿ Apiocephalus yangmingae sp. nov.

Taxon classificationAnimaliaColeopteraCerambycidae

D950883A-CD8C-5F7E-BE49-4D9B1328B602

https://zoobank.org/90F9D9C3-13A5-4195-83A3-E94B41D40BD4

**Figure 3.**
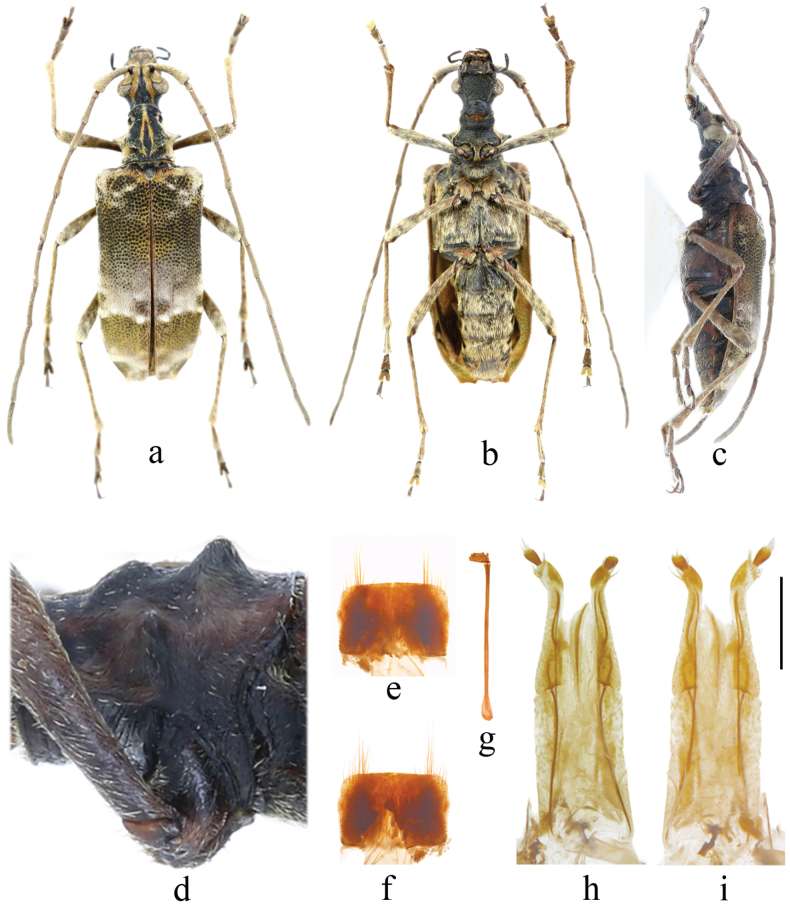
Holotype of *Apiocephalusyangmingae* sp. nov., female **a–d** habitus of adult **e–i** terminalia **a, e, i** dorsal view **b, f, h** ventral view **c** lateral view **d** lateral view of the pronotum **e, f** sternite VIII **g** tignum **h, i** ovipositor **c, d** taken after remounted. scale: 1 mm (**e–i**).

Chinese common name: 阳明头花天牛 Fig. 3

#### Description.

***Holotype*, female.** Body length 13.5 mm (from the front of mandible to elytral apex), humeral width 4.5 mm. Body dull yellowish-brown to black, clothed with greyish-white, greyish-yellow to golden yellow pubescence. ***Head*** black, clothed with greyish-yellow pubescence, which is thicker and denser on frons, upper half of genae and sides of the vertex; pubescence on sides of the vertex predominantly golden, forming two longitudinal stripes from antennal insertions to occiput. ***Pronotum*** black, clothed with dense greyish-yellow pubescence on sides (including lateral spines) and provided with a golden yellow longitudinal pubescent stripe on either side of midline, connected anteriorly but not reaching basal margin posteriorly; the remainder clothed with rather sparse greyish-yellow pubescence. ***Scutellum*** blackish-brown, clothed with greyish-white pubescence. ***Elytra*** dark brown, clothed with rather thin greyish-yellow to golden pubescence, and adorned with patches of relatively dense greyish-white pubescence; each elytron with a narrow transverse band at extreme base (golden near scutellum), extending inwards slightly behind humerus, then bending obliquely forward, forming an irregular ring; a broad transverse band behind middle, of which anterior boundary indistinct, posterior edge with five faintly visible spots of dense pubescence, each side containing a region of less greyish-white pubescence; an irregular transverse band before apex and a tuft of greyish-white setae at marginal angle. ***Underside*** mostly clothed with dense and non-uniformly greyish-yellow pubescence, prosternum nearly glabrous except for intercoxal process; abdominal ventrites with a glabrous blackish-brown spot on each side. Legs mostly dull yellowish-brown, clothed with greyish-yellow pubescence.

***Head*** prolonged and distinctly narrower than pronotum, featuring a median groove from antennal insertions to upper eye lobes; eyes obviously protruding outward; labrum twice as wide as long, emarginate on apical margin; genae slightly shorter than eye diameter; terminal segment of maxillary palpus prolonged spindle-shaped, with truncate apex. Antennae slightly longer than body, with the distal segment extending beyond the elytral apex; scape long, nearly reaching apical margin of pronotum, and strongly thickened apically; antennomere 3 about as long as antennomere 4 and shorter than antennomere 5. ***Pronotum*** slightly shorter than width between lateral spines; apical margin remarkably narrower than basal margin; sides conspicuously constricted at about apical fifth, with lower portion near apical edge distinctly expanded laterally; median spines well developed, blunt apically; disc coarsely and shallowly punctate, with four protuberances, of which the anterior pair before middle are rather low, the posterior pair behind middle are strongly raised, and also with several vague short longitudinal ridges near centre. ***Scutellum*** ligulate, slightly concave apically. ***Elytra*** about two times as long as width across humeri, subparallel-sided, and arcuately convergent to apices on apical fifth; apices truncate, squarish at outer angles, rounded at inner angles; disc quite uneven, especially at base and apex; surface with extreme base granular-punctate, basal half deeply and closely punctate, apical half more shallowly and sparsely punctate; each elytron provided with an indistinct longitudinal costa. ***Prosternal process*** strongly arcuate, narrowed between procoxal cavities, then steeply sloped backwards and dilated apically. ***Legs*** long and slender; metafemora not reaching the elytral apex; metatarsomere 1 shorter than the remaining combined.

**Male.** Unknown.

#### Type material.

***Holotype***, **China** • female (YZU); Shaanxi, Yangxian (洋县), Huayang town (华阳镇), Hongshiyao village (红石窑村); 33°38'24"N, 107°29'24"E; alt. 1317 m; 8 May 2018; Guanglin Xie leg.

#### Distribution.

China: Shaanxi.

#### Etymology.

The new species is named in honour of Ms Zhou Yangming, the author’s mother, a traditional Chinese woman known for her gentle nature, intelligence, manual dexterity and family values. She passed away in 2022 while the author was visiting the Natural History Museum in London.

#### Diagnosis.

This new species can be distinguished from its congeners by the more exposed dorsum of the head and pronotum, both featuring golden yellow longitudinal stripes, more elongate pronotal lateral spines, and different elytral markings.

#### Remarks.

The new species was collected on an unknown flower, suggesting a flower-visiting habit.

### ﻿Key to the known species of *Apiocephalus* Gahan

**Table:** 

1	Head and pronotum mostly clothed with greyish-white to greyish-yellow pubescence, without longitudinal stripes; elytra mostly clothed with greyish-white to yellowish pubescence, mottled with black or dark brown spots	**2**
–	Head and pronotum mostly glabrous, with golden yellow longitudinal stripes; elytra provided with irregular greyish-white pubescent bands on base, behind middle and apices	***A.yangmingae* sp. nov.**
2	Pronotum thinly clothed with greyish-white pubescence; elytra mostly clothed with greyish-white to greyish-yellow pubescence, with glabrous black spots fusing into large patches before middle	***A.punctipennis* Gahan**
–	Pronotum densely clothed with greyish-white pubescence; elytra mostly clothed with greyish and tawny pubescence, scattered with dark brown pubescent spots, with apical fourth clothed with dense white pubescence	***A.licheneus* Gahan**

## Supplementary Material

XML Treatment for Apiocephalus

XML Treatment for Apiocephalus punctipennis

XML Treatment for Apiocephalus licheneus

XML Treatment for Apiocephalus yangmingae

## ﻿Additional information

### Author ORCIDs

Guanglin Xie https://orcid.org/0000-0002-1021-9060

### Data availability

All of the data that support the findings of this study are available in the main text.
